# Mechanical stress‐induced autophagy is cytoskeleton dependent

**DOI:** 10.1111/cpr.13728

**Published:** 2024-08-18

**Authors:** Lin Liu, Wei Zheng, Yuhui Wei, Qian Li, Nan Chen, Qinglin Xia, Lihua Wang, Jun Hu, Xingfei Zhou, Yanhong Sun, Bin Li

**Affiliations:** ^1^ Key Laboratory of Laboratory Medicine, Ministry of Education of China, Zhejiang Provincial Key Laboratory of Medical Genetics, School of Laboratory Medicine and Life Sciences Wenzhou Medical University Wenzhou China; ^2^ Shanghai Institute of Applied Physics, Chinese Academy of Sciences University of Chinese Academy of Sciences Shanghai China; ^3^ The Interdisciplinary Research Center, Shanghai Synchrotron Radiation Facility Shanghai Advanced Research Institute, Chinese Academy of Sciences Shanghai China; ^4^ Frontiers Science Center for Transformative Molecules and National Center for Translational Medicine Shanghai Jiao Tong University Shanghai China; ^5^ School of Chemistry and Materials Sciences Shanghai Normal University Shanghai China; ^6^ Institute of Materiobiology, College of Science Shanghai University Shanghai China; ^7^ Department of Microelectronic Science and Engineering, School of Physical Science and Technology Ningbo University Zhejiang China

## Abstract

The cytoskeleton is essential for mechanical signal transduction and autophagy. However, few studies have directly demonstrated the contribution of the cytoskeleton to mechanical stress‐induced autophagy. We explored the role of the cytoskeleton in response to compressive force‐induced autophagy in human cell lines. Inhibition and activation of cytoskeletal polymerization using small chemical molecules revealed that cytoskeletal microfilaments are required for changes in the number of autophagosomes, whereas microtubules play an auxiliary role in mechanical stress‐induced autophagy. The intrinsic mechanical properties and special intracellular distribution of microfilaments may account for a large proportion of compression‐induced autophagy. Our experimental data support that microfilaments are core components of mechanotransduction signals.

## Graphical abstract

Microfilaments are an essential element, whereas microtubules play a relatively small role as an auxiliary factor in mechanical stress‐induced autophagy. The deformation of the elastic cortical microfilaments caused by compression accounts for a large proportion of force‐induced autophagy.

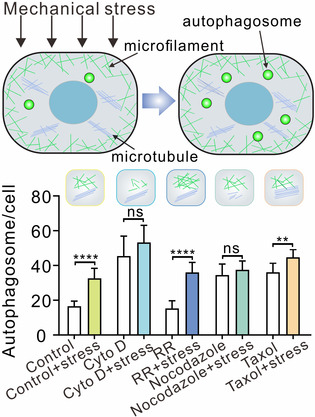

## 1 INTRODUCTION

Macroautophagy (hereinafter autophagy) is a well‐recognized degradative process in which damaged, degenerated, or aged cytoplasmic proteins and organelles are packaged into double membranes and transported to the lysosomes for digestion and degradation. It is indispensable for many cellular processes, especially cellular homeostasis and survival.^1^, ^2^, ^3^ Various physiological and pathological stresses induce autophagy, including adverse environment, starvation, endoplasmic reticulum stress, hypoxia, DNA damage, and pathogen infection.^4^, ^5^, ^6^, ^7^, ^8^ Internal and external mechanical stimuli, such as gravity, impact force, blood flow shear force, intercellular extrusion force, and muscle tensile force, also effectively induce autophagy.^9^, ^10^ Although mounting studies have demonstrated that mechanical stimulation in the cellular environment can effectively induce autophagy, it is unclear how the mechanical stimuli are perceived and converted into intracellular autophagy signals.

Mechanotransduction is a fundamental biological process through which cells detect mechanical changes and convert them into intracellular signals. Any external force on cells can result in direct coupling to force‐sensitive channels by cellular structures, including the cytoskeleton, from which a mechanical signalling pathway is formed.^11^, ^12^ Recent studies have confirmed that the cytoskeleton is involved in force‐feedback mechanisms related to cellular mechanosensation^13^, ^14^ and even serves as a core component of mechanotransduction.^15^ For example, mechanosensitivity was weak in a cytoskeleton‐free cell membrane.^16^ It has also been reported that the cytoskeleton functions mainly via linear structures with a high elastic modulus.^17^ We have previously shown that the cytoskeleton plays a vital role in maintaining the cellular elastic modulus, a significant physical property related to resistance to changes under mechanical stress and demonstrated that acute shear stress, nano‐Newton compression, and acupunctural stimulation could effectively induce autophagy in cultured cells and living animals.^18^, ^19^, ^20^, ^21^ Based on the above evidence, we speculated that the cytoskeleton is an essential structure for mechanotransduction and plays an important role in mechanical force‐induced autophagy.

In this study, we investigated the effects of the cytoskeleton on mechanical force‐induced autophagy at the cellular level. Using fluorescent labelling techniques and western blotting, we first determined the combination of force and time of compression required to induce autophagy. Subsequently, we assessed the roles of microfilaments (MF) and microtubules (MT), two major cytoskeletal components involved in mechanical stress‐induced autophagy, to determine whether they play essential roles or act as auxiliary elements. We recorded the dynamic changes in autophagosomes induced by in situ compression to further test the role of MF. Finally, we characterized cellular elasticity using atomic force microscopy (AFM). Our approach allowed us to evaluate not only the required cytoskeletal elements in force‐induced autophagy but also the roles of their inherent physical properties in autophagy.

## 2 MATERIALS AND METHODS

### 2.1 Cell culture

HeLa, PC12, and N2A cells were purchased from the Stem Cell Bank/Stem Cell Core Facility (SIBCB, CAS, China) and cultured as in our previous studies.^19^ Briefly, HeLa and N2A cells were maintained in Modified Eagle's Medium, while PC12 cells were maintained in Dulbecco's Modified Eagle's Medium, containing 10% foetal bovine serum, 1% L‐glutamine, and 1% penicillin/streptomycin at 37°C in a humidified atmosphere containing 5% CO_2_. All culture media were obtained from Gibco (Waltham, USA).

### 2.2 Plasmids transfection

The pBABEpuro GFP‐LC3 plasmid was obtained from Addgene. HeLa‐EGFP‐LC3, N2A‐EGFP‐LC3, and PC12‐EGFP‐LC3 cells were constructed as described.^19^ Transfection with Lipofectamine 3000 (Invitrogen) was performed according to the manufacturer's instructions. The cells were cultured in G418 Medium (Selleck Chemicals, Houston, TX). High‐expressing cell clones were identified using a fluorescence microscope.

### 2.3 Treating cells with drugs

Cells were treated with drugs as reported previously.^18^ Briefly, HeLa cells were treated with cytochalasin D (Cyto D) (Selleck Chemicals, Houston, TX), ruthenium red (RR) (Apex BIO, Houston, TX), nocodazole, and Taxol (Selleck Chemicals) with the final working concentration of 0.25, 15, 2, and 10 μM for 2 h, respectively. The cells were washed three times with 3 ml of 1× PBS at room temperature.

### 2.4 MF and MT staining

The MF and MT of HeLa‐EGFP‐LC3 cells were stained with actin‐tracker red‐555 probe and tubulin‐tracker red probe (Beyotime, Beijing, China), respectively. The detailed experimental steps were performed according to the manufacturer's instructions.

### 2.5 Fluorescence imaging

Fluorescence images were acquired using a laser confocal fluorescence microscope (Nikon A1R, Japan) equipped by a 60× (NA1.45) and a 480/520 nm laser line. In situ fluorescence images of autophagy dynamics were captured using a fluorescence microscope **(**Leica DMI 3000 B, Germany) by oil‐immersion 63 × (NA1.4) with a 480 nm laser line.

### 2.6 Western blotting analysis

Western blotting analysis was conducted as previously described.^4^ Briefly, the total protein of samples was quantified by a BCA protein kit (Beyotime), separated on a 12% sodium dodecyl sulphate‐polyacrylamide gel (SDS‐PAGE), and transferred onto 0.45 μm polyvinylidene difluoride membranes (Millipore, Billerica, MA). After blocking with 5% skimmed milk, the membranes were incubated with p62, Beclin‐1, LC3I/II, and GAPDH (Cell Signalling Technology, Danvers, MA) primary antibodies overnight at 4°C, and then HRP‐secondary antibodies (Beyotime). Finally, the immunoblots were analysed using enhanced chemiluminescence reagents (Bio‐Rad, Hercules, USA).

### 2.7 Macroscopic compression on cell

The compressive forces were applied to different groups of cells (treated or not treated with drugs), as the previously described.^22^ Briefly, the cells in the culture dish were placed from bottom to top with 1% agarose (Sangon Biotech, Shanghai, China) film, a plastic cover with holes, a cover glass, and weights. The weight could be switched according to the compressive force required. After 5 min of compression, the above four materials were removed and the cells were imaged through fluorescence imaging.

### 2.8 Microscopic compression on cell

After the cells' fluorescence images were obtained, the DNP‐S10 tip (spring constant of 0.35 N/m, tip radius of 10 nm) was located on the top central of the cells. Typically, a 5 nN force was applied to scan the 45 μm × 45 μm cell area at 1 Hz, for 45 min. Fluorescence/AFM images were obtained before and after the nanomechanically induced autophagy was completed. All AFM/fluorescence microscope experiments were performed in Leibovitz's‐15 cell culture medium (Gibco) at 37°C and 40%–50% humidity.

### 2.9 Elastic measurement

The Young's modulus of HeLa cells was measured using a Bioscope Resolve AFM (Bruker, USA) in combination with the fluorescence microscope. The cell elastic characteristics were measured using spherical probe with diameter of 2.5 μm (Novascan Technologies, USA) as described previously.^19^ Data were processed using Bruker data processing software (NanoScope Analysis 2.0).

### 2.10 Data analysis

Fluorescence intensity was analysed by Image J software. All obtained data and figures were generated using GraphPad Prism (version 8.0.3, GraphPad Software Inc., La Jolla, CA). Statistical significance was tested using Student's *t*‐test or one‐way ANOVA. All data are represented as mean and SEM.

## 3 RESULTS

### 3.1 Induction of autophagy by mechanical stress in human cells

**FIGURE 1:**
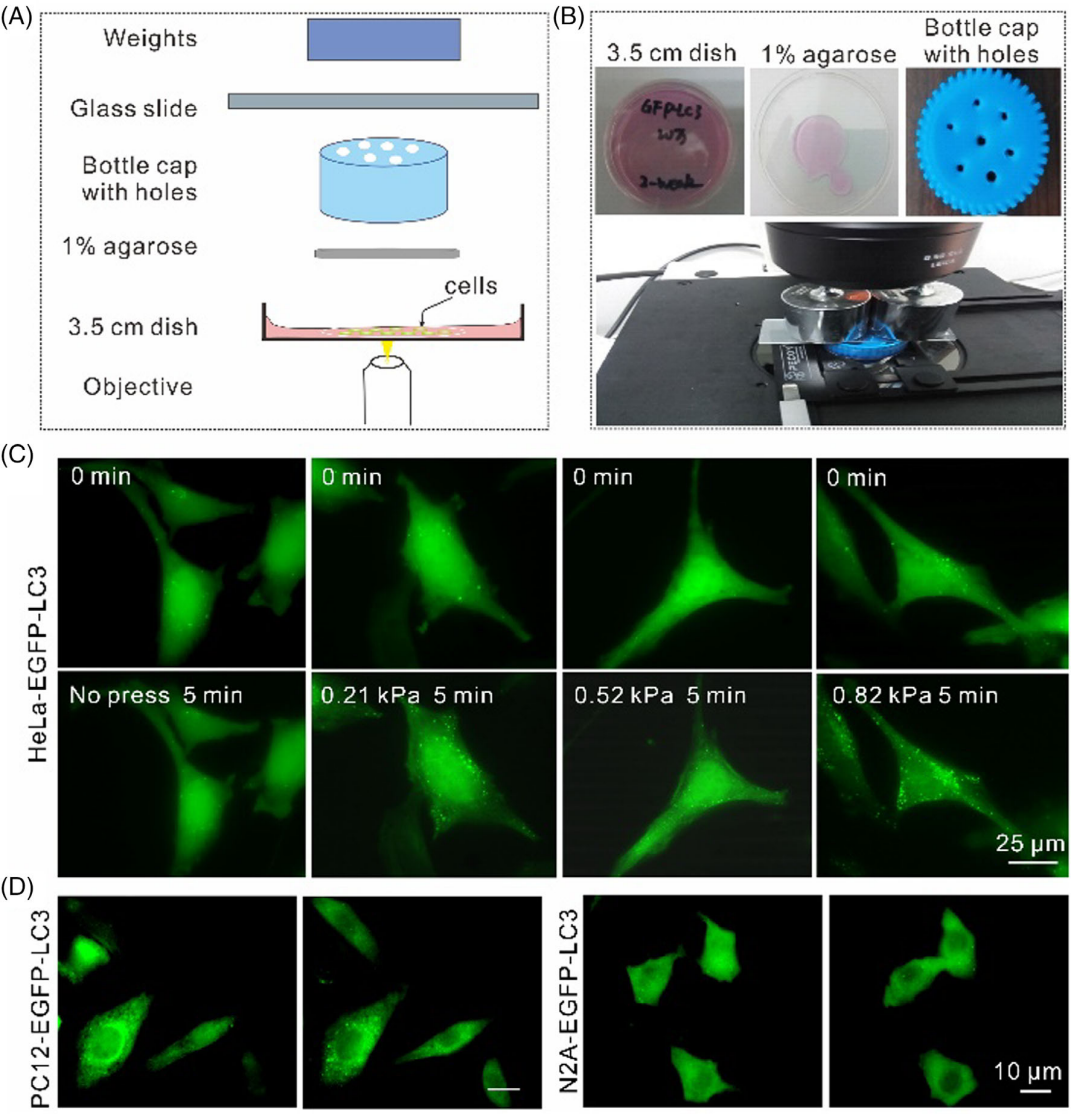
Compressive force induces autophagy. (A–B) An experimental set up is used to exert force upon cells. The arrangement of components is shown in (A) the expanded and (B) the assembled views.^22^ (C) Changes in autophagy in HeLa‐EGFP‐LC3 cells after 5 min of different forces. All images were captured in situ using a fluorescence microscope (scale bar: 25 μm). (D) Mechanical stress‐induced autophagy in N2A‐EGFP‐LC3 and PC12‐EGFP‐LC3 cells after 5 min of 0.52 kPa. All images were captured in situ using a fluorescence microscope (scale bar: 10 μm).

We estimated the effect of compression on autophagy in cells expressing the EGFP‐labelled MT‐associated protein 1 light chain 3 (LC3), an essential autophagy protein that is processed into LC3‐I and subsequently converted to LC3‐II if localized to the autophagosomal punctate membrane.^23^ This is a widely recognized method for autophagosome labelling and identification. The number of EGFP‐LC3‐II puncta represents autophagic activity within cells, and the dynamic autophagic process can be monitored by live cell imaging. To monitor autophagy induced by mechanical stress, we used a facile experimental setup to apply mechanical force to the cells. With reference to previously reported methods, cells in a culture dish were mounted on a fluorescence microscope and gently pressed down with a weight from above.^22^ The experimental setup and a simple diagram are shown in detail in Figure 1A,B. The morphology of the cytoskeleton and the number of autophagosomes were examined directly before/ and after the weight was moved. We applied force (0.21, 0.52, and 0.82 kPa) to the HeLa‐EGFP‐LC3 cells for 5 min. Autophagosomes (puncta) were induced using various compressive forces (Figure 1C). Micrographs show an increase in puncta under mechanical force relative to untreated cells. These findings were consistent with previous results, indicating that pressures of 0.07, 0.25, and 1.15 kPa can trigger autophagy.^22^ Additionally, we examined cellular activities using a moderate compressive force. The number and morphology of the cells in the culture dish became slightly altered after continuous compression (0.52 kPa) for 5 min (data not shown), indicating that less cellular damage occurred under this condition. Finally, we screened several human cell lines, including HeLa‐EGFP‐LC3, N2A‐EGFP‐LC3, and PC12‐EGFP‐LC3, to study the efficiency of compression‐induced autophagy. As shown in Figure 1D, the HeLa‐EGFP‐LC3 cell showed a greater response to mechanical stress (0.52 kPa) than the N2A‐EGFP‐LC3 and PC12‐EGFP‐LC3 cell lines and applying force for 5 min induced new puncta within the cells in our experimental system. Thus, HeLa‐EGFP‐LC3 cell lines were used in subsequent experiments, with a compression force of 0.52 kPa on top of the cells in the culture dish for 5 min.

### 3.2 MF is required for mechanical stress‐induced autophagy

**FIGURE 2:**
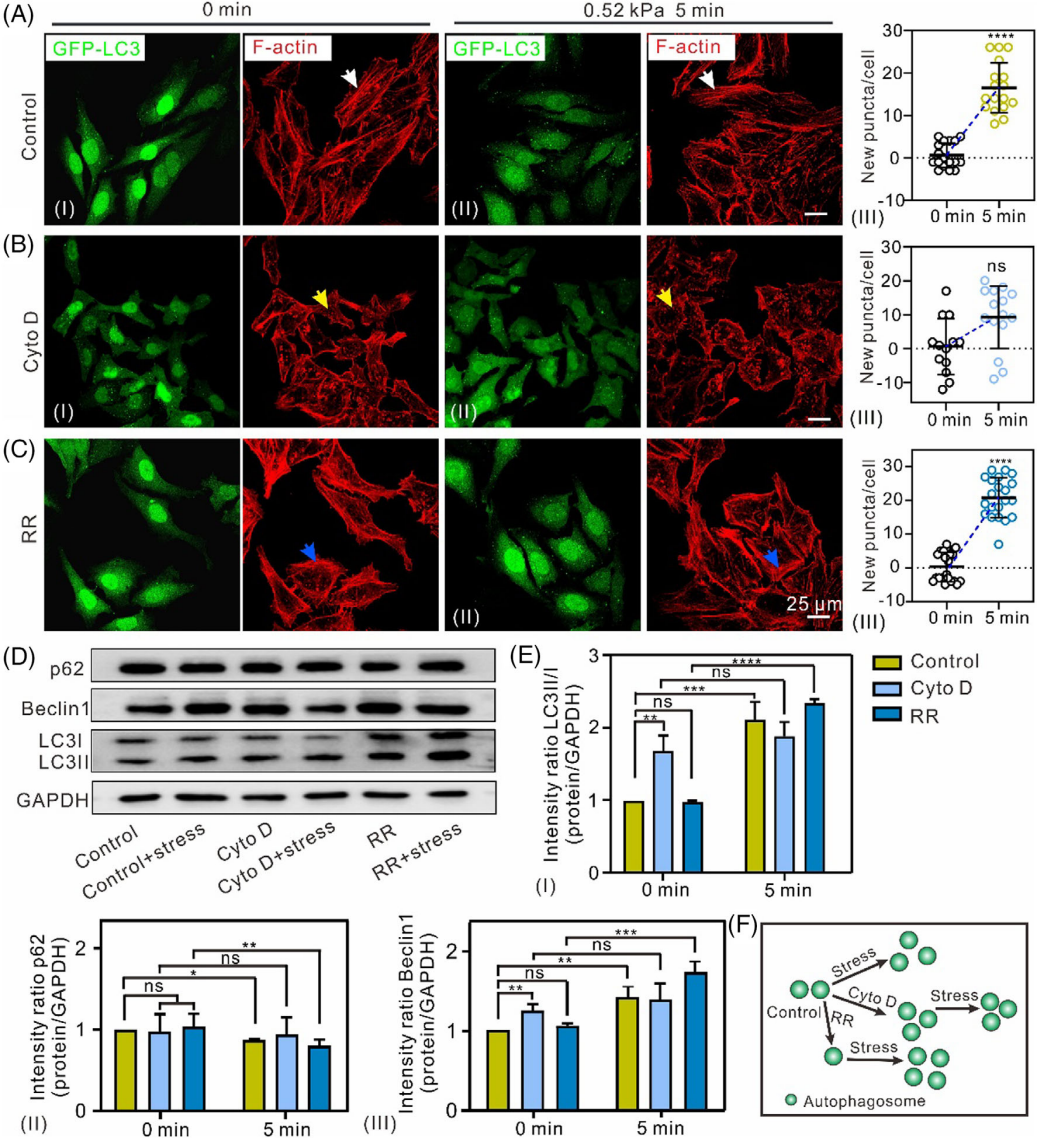
Role of MFs in mechanical stress induced‐autophagy. (A–C) Changes in MT morphology and the number of punctate structures (arrows) unloading (I) and loading (II) stress in three groups of HeLa‐EGFP‐LC3 cells. (III) New puncta in individual cells. (D) Western blotting analysis shows the changes in autophagy levels in three group cells induced by compression. (E) The expression levels of the LC3II/I, p62 and Beclin 1 proteins were quantitatively analyzed (n ≥ 3, ns, not significant, *p < 0.05, **p < 0.01, ***p < 0.001 ****p < 0.0001). (F) The schematic diagram of autophagosome changes under difference MF conditions.

To determine the role of MF in mechanically induced autophagy, HeLa‐EGFP‐LC3 cells were stained with Actin‐tracker red‐555. For HeLa‐EGFP‐LC3 or HeLa cells, the MFs were distributed as continuous and dense filaments (Figures 2A–I, II (white arrow) and S1), while compression induced a significant increase in the number of autophagosomes (Figure 2A‐III). For HeLa‐EGFP‐LC3 or HeLa cells pretreated with Cyto D, a chemical commonly used to disassemble MFs,^24^ shorter rather than longer filaments were detected within the cells (Figures 2B–I, II (yellow arrow) and S1); no new puncta appeared in the cells (Figure 2B‐III) after the force was applied, suggesting that autophagy induced by mechanical stress may require intact MF. The apparent increase in puncta induced by Cyto D treatment compared to the groups not treated with Cyto D could be explained by the role of MFs in autophagosome movement and autophagosome‐lysosome fusion^25^, ^26^; the degradation of MF structures leads to reduced autophagosome degradation, resulting in increased puncta in cells. For HeLa‐EGFP‐LC3 or HeLa cells pretreated with RR, a chemical applied to promote the polymerization of MFs,^18^ the number of longer MF increased slightly (Figures 2C–I, II (blue arrow) and S1), while a significant increase in puncta was observed after mechanical stress (Figure 2C‐III). The decreased number of puncta after treatment with RR (Figure 2C‐III) may be due to increased puncta movement and lysosome fusion caused by MF polymerization.^25^ Importantly, we found that the increase in puncta was more obvious in the RR‐pretreated than in the control cells (Figure 2A‐III, C‐III), suggesting that increasing the polymerization of MF could effectively promote the sensitivity of compression‐induced autophagy (Figure 2C), leading to a substantial increase in de novo autophagosome formation. To verify the role of MF in compression‐induced autophagy, we applied nano‐Newton compression to individual Cyto D‐pretreated HeLa‐EGFP‐LC3 cells using an AFM probe. These results were consistent with those of bulk experiments, and no significant increase in new autophagosomes was observed (Figure S2). Western blotting analysis revealed that mechanical stress‐induced changes in autophagy is discrepant for the cells pretreat with Cyto D and RR: an increasing of LC3II/I and Beclin1 with decreasing of p62 in control group, an increased LC3II/I but no apparent alteration in p62 and Beclin1 in Cyto D treated‐cells, and a significant increase of LC3II/I and Beclin1, as well as a significant decrease of p62 in RR treated‐cells (Figure 2D–F). The little change in autophagy level in Cyto D pretreated‐cells and obvious increase in autophagy levels in the RR pretreat‐cells suggested that promoting MF polymerization increased the sensitivity of cells to mechanical force‐induced autophagy, whereas MF disruption reduced the response of cells to compression. These results indicated that intact MF elements are required for mechanical stress‐induced autophagy.

### 3.3 Role of MT in mechanical stress‐induced autophagy

**FIGURE 3:**
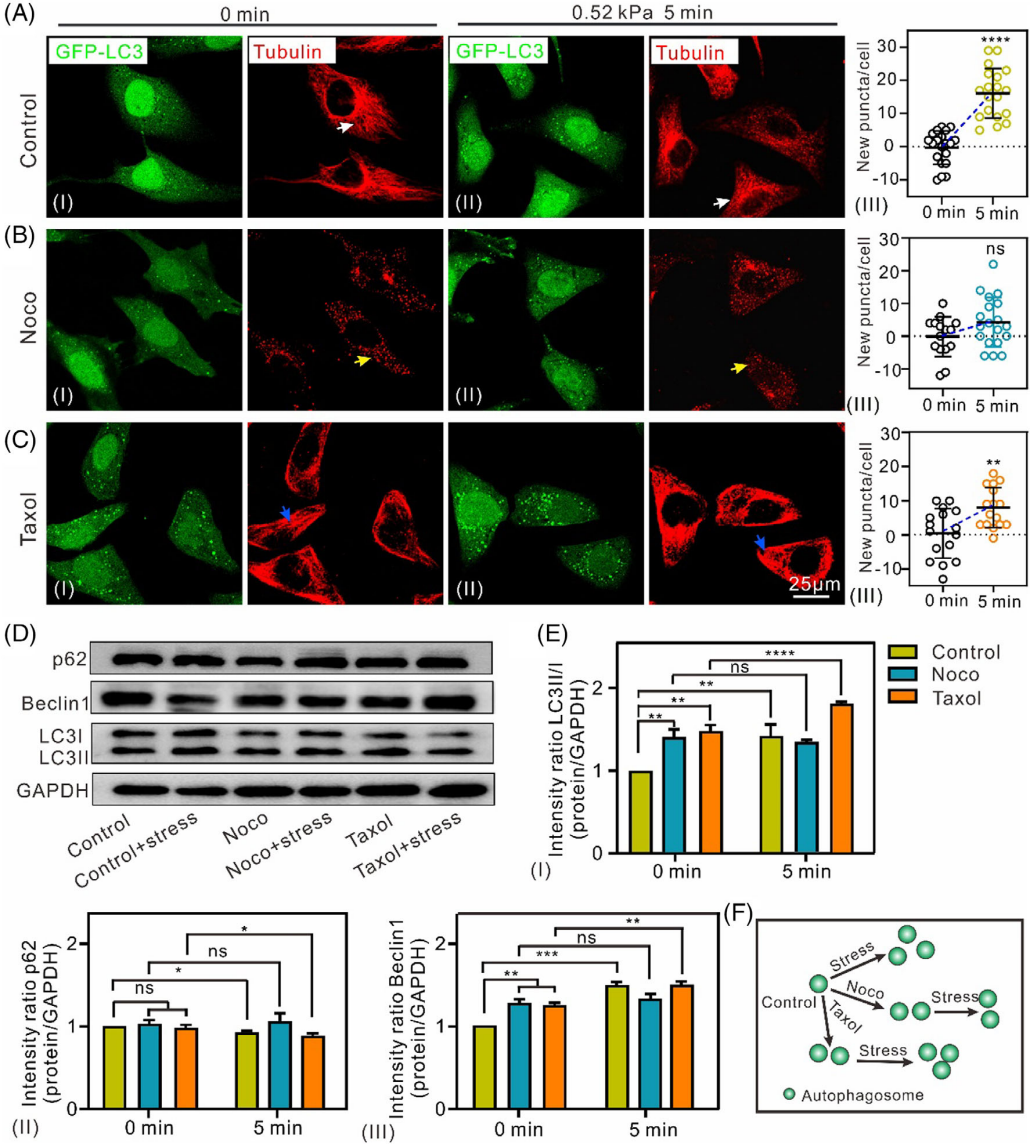
Role of MTs in mechanical stress‐induced autophagy. (A–C) Changes in MT morphology and the number of punctate structures (arrows) unloading (I) and loading (II) stress in groups of HeLa‐EGFP‐LC3 cells. (III) New puncta in individual cells. (D) Western blotting analysis shows the changes in autophagy levels in three group cells induced by compression. (E) The expression levels of the LC3II/I, p62 and Beclin 1 proteins were quantitatively analysed (*n* ≥ 3, ns, not significant, *p < 0.05, **p < 0.01, ***p < 0.001, ****p < 0.0001). (F) The schematic diagram of autophagosome changes under difference MT related drugs and stress.

To evaluate the role of MTs in compression‐triggered autophagy, cells were stained with Tubulin Tracker Red. In HeLa‐EGFP‐LC3 or HeLa cells, MTs were distributed as continuous and dense filaments (Figures 3A–I, II [white arrow] and S3), and compression induced a significant increase in the number of autophagosomes (Figure 3A‐III). For cells pretreated with nocodazole, a chemical commonly used to disassemble MTs,^27^ there was a slight increase in autophagosomes (Figure 3B–I‐II) accompanied by fractured and sparse MT (Figures 3B–I, II (yellow arrow) and S3). As expected, no apparent autophagosomal changes were observed under compression (Figure 3B‐III). For the Taxol‐pretreated HeLa‐EGFP‐LC3 or HeLa cells, a chemical commonly used to stabilize MTs,^28^, ^29^ there was an increase in autophagosomes (Figure 3C–I‐III) accompanied by long and dense MT adjacent to the nucleus (Figures 3C–I, II [blue arrow] and S3). Unexpectedly, only slight changes in autophagosomes were detected after compression was applied to the cells (Figure 3C). These results imply that MT is likely dispensable under certain force‐triggered autophagosome formation conditions. Western blotting analysis demonstrated that mechanical stress‐induced changes in autophagy were discrepant for the cells pretreated with Noco and Taxol: a significantly increased of LC3II/I and Beclin1, as well as a decreasing p62 in the control group, almost no change in LC3II/I, Beclin1, and p62 in the pretreat‐Noco group, an increase in LC3II/I and Beclin1, as well as a decreasing in p62 in the pretreat‐Taxol group (Figure 3D–F). The increase in autophagy levels in the pretreat‐Taxol and the control groups were similar. These results suggest that MTs participate less in compression‐induced autophagy under certain conditions, although previous studies have reported that MTs mediate autophagosome formation and fusion of autophagosomes with endosomes.^25^, ^26^

Transfection of EGFP‐LC3 plasmids may affect the microtubules themselves, which is likely to affect detected fluorescence signals of autophagy. The results of immunofluorescence staining showed less interference with autophagy signals, LC3II and p62, after transfection of EGFP‐LC3 plasmids into HeLa cells (Figure S4), implying that transfection of EGFP‐LC3 plasmids has little effect on autophagy in our experimental system.

### 3.4 Dynamic changes of autophagosomes induced by compression in situ

**FIGURE 4:**
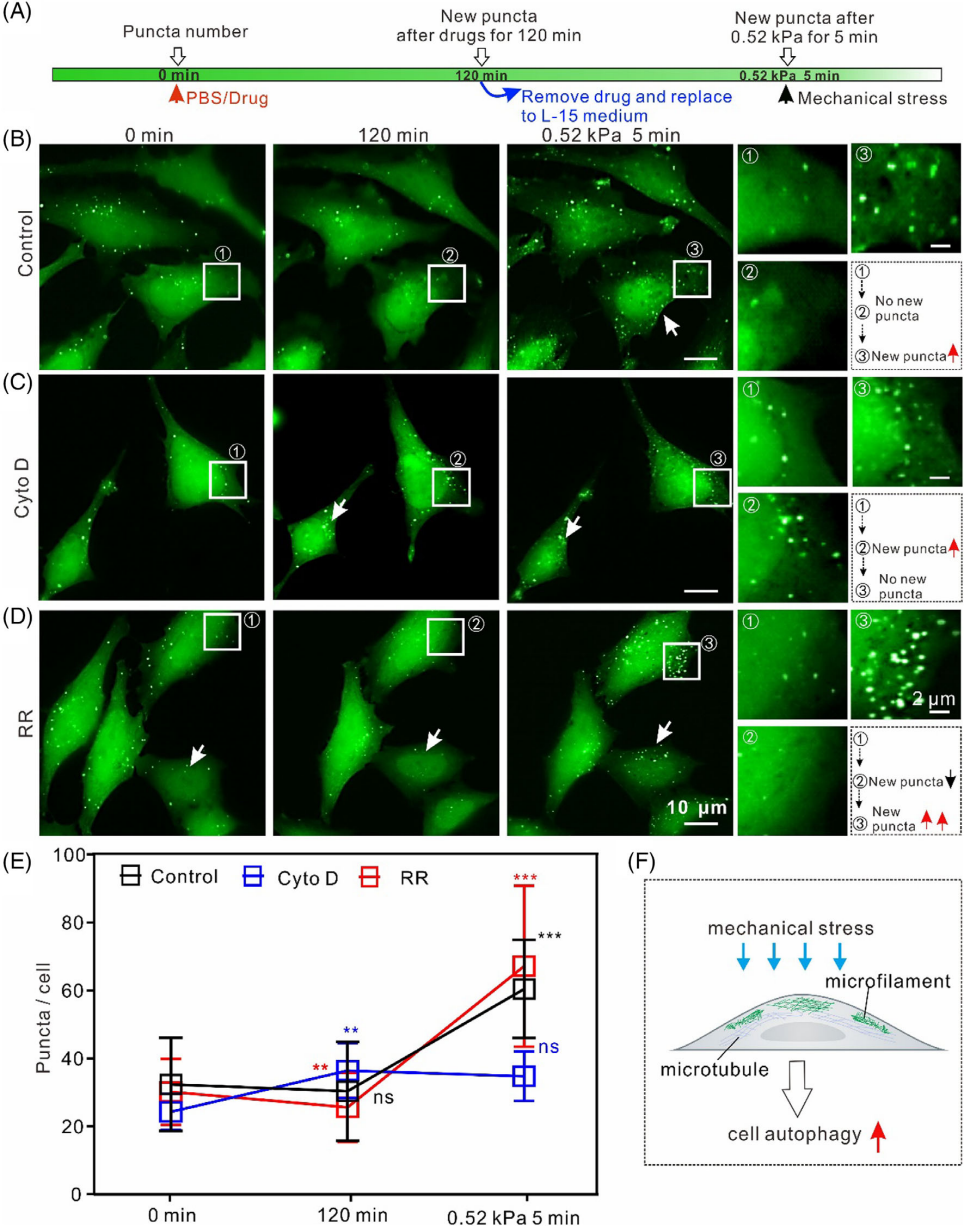
The autophagosome dynamics induced by compression in situ. (A) Schematic illustration of the change in EGFP‐LC3 puncta after drug treatments and compression. (B–D) Dynamics of autophagosomes under compression in the three groups of cells. The magnified region in the white square region shows the detailed dynamic change in autophagosomes. White arrows indicate cells with autophagosome change. Black arrow indicates decreased autophagosomes; red arrow indicates increased autophagosomes. (1) 0 min; (2) drug‐treatment for 120 min; (3) 0.52 kPa for 5 min; (E) Average number of puncta in different drug‐induced cells (*n* = 5, ns, not significant, **p < 0.01, ***p < 0.001). (F) The cartoon diagram illustrates the compression‐induced autophagy relative to the spatial distribution of MF and MT in cells.

To further elucidate the role of MF in mechanical stress‐mediated autophagy, dynamic changes in autophagosomes were observed in situ using a fluorescence microscope. Figure 4A depicts the experimental procedures for applying the drug and mechanical force applied to the cells. Firstly, the three groups of HeLa‐EGFP‐LC3 cells were treated with PBS/Cyto D/RR, for 120 min; then, these reagents were replaced with Leibovitz's‐15 cell culture medium. Finally, mechanical force was applied using 0.52 kPa for 5 min. Figure 4B shows the basal dynamic state of autophagosomes in control HeLa‐EGFP‐LC3 cells. The number of puncta in different parts of the cell fluctuated slightly; there was no a significant change in the total number of puncta at 120 min. Mechanical stress effectively induced the formation of new autophagosomes (Figure 4B [white arrow], enlarged image of the white square). This phenomenon differed significantly in cells pretreated with the drugs. Autophagosomes increased in Cyto D‐treated cells at 120 min, and mechanical stress almost did not induce the formation of new autophagosomes (Figure 4C (white arrow), enlarged image in the white box). In contrast, RR‐treated cells showed a decrease in the number of autophagosomes at 120 min, whereas mechanical stress significantly increased the autophagosome number (Figure 4D (white arrow), enlarged image in the white box). These distinct dynamic processes further demonstrate that MFs are an essential element in autophagy induced by mechanical stress. We quantitatively evaluated the effects of MF on the dynamics of autophagy. As shown in Figure 4E, Cyto D‐ and RR‐pretreated cells showed an increase and decrease in autophagosomes by approximately 50% and 15%, respectively, whereas there was little change in the number of autophagosomes in the control group at 120 min. However, the number of autophagosomes increased significantly in RR‐pretreated cells, reaching up to approximately 150% after mechanical force loading. These results strongly suggested that autophagosome formation induced by mechanical stress requires MF structures (Figure 4F). We also tracked autophagosome dynamics in Noco‐treated cells. As expected, the number of autophagosomes within cells did not change significantly under the same compression conditions (Figure S5). These results further indicate that although MT is involved in mechanical stress‐triggered cell autophagy, MF plays a crucial role.

### 3.5 Correlation between the change in autophagy and the elastic modulus

**FIGURE 5:**
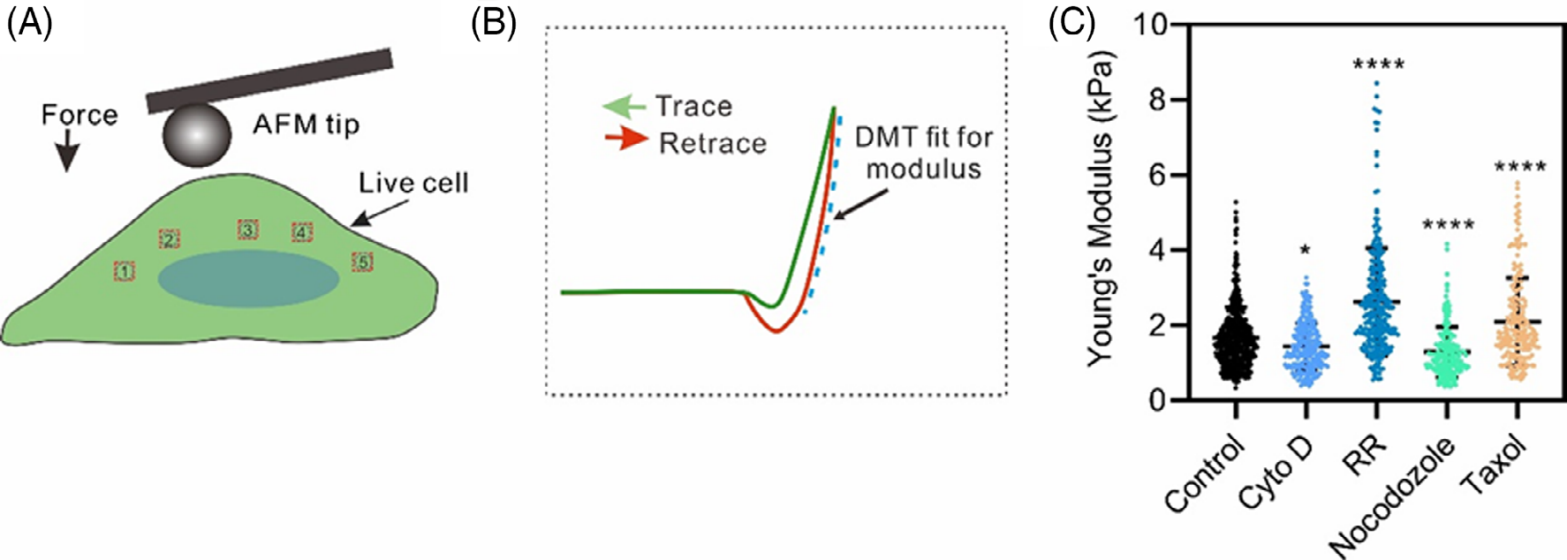
Measured elasticity of HeLa‐EGFP‐LC3 cells subjected to different chemical treatments. (A) Schematic diagram of cellular elasticity measurement using AFM. Five randomly positioned sites in the centre of each cell were used for measurement. (B) Schematic force‐distance curve of AFM. Young's moduli obtained using the data for retrace curve (dashed blue) fitting in DMT mode. (C) Measured Young's modulus of the cells. Statistical data were obtained from five independent experiments with at least five cells per group in each experiment (*n* >30 cells, *p < 0.05, ****p < 0.0001).

To explore the correlation between the change in autophagy and the inherent elasticity, the elastic modulus of the cells was measured using AFM.^19^ The force curves were obtained using an AFM tip at five random points on the cell surface (Figure 5A,B). The representative force curves are shown in Figure S6. The measured cellular elasticity shows that Young's modulus of cells decreased or increased significantly after Cyto D or RR treatment, respectively, and after treatment with nocodazole and Taxol (Figure 5C), indicating that MF and MT play a primary role in maintaining cell elasticity. By analysing the correlation between the elastic modulus (Figure 5C) and the change in autophagy (Figures 2 and 3), we found that an increase in the elastic modulus caused by MF polymerization corresponded to enhanced autophagy. MT polymerization also increased cell elasticity; however, the increase in autophagy was reduced, supporting the key role of MF elasticity in mechanically induced autophagy.

## 4 DISCUSSION

We investigated the role of the cytoskeleton in mechanical stress‐induced autophagy using chemical agents and found an increase in compression‐induced autophagy when MF were polymerized, but a decrease in autophagosome changes when MT were polymerized. We conclude that MF, rather than MT, play a major role in mechanical stress‐induced autophagy. We inferred that this may be partly due to differences in the intrinsic mechanical properties and spatial distribution of MF and MT within the cells.

First, as two basic cytoskeletal polymers of the three major structural components, MF and MT are highly ordered scaffolds that exist as networks that shape and support cells.^30^ However, owing to the differences in the composition and arrangement of scaffold‐like protein structures, their mechanical stiffness differs.^18^, ^31^, ^32^ Although individual MFs are much less rigid than MTs,^33^ the highly organized MF network still has excellent mechanical properties and largely determines the shape and elastic properties of cells.^18^, ^29^, ^34^, ^35^, ^36^ These elastic MF network structures physically connect the cell to the external environment through the cell membrane and may be more sensitive to changes in the external environment than MT networks. Therefore, we investigated the effects of drugs on cellular elasticity, and analysed the correlation between cellular elasticity and autophagy triggered by mechanical forces.

In theory, an increase in the Young's modulus of a cell implies that the deformation of the cell is reduced under the same compressive force. Why the increased cellular elasticity is accompanied by an increased effect on trigger autophagy? We believe that the polymerization of MF makes the cytoskeletal network denser, and the denser network architecture has multiple sites for sensing, transmitting, and transducing forces, thus it has a strong force‐autophagy signal transformation ability. These factors may enhance the sensitivity of cells to mechanical forces, such that small cellular deformations can effectively induce autophagy. However, this is not the case for MT. In particular, MT stabilization increases the cellular Young's modulus but leads to a decrease in the number of stress‐induced nascent autophagosomes. This result is similar to the starvation‐induced autophagy reported by Geeraert et al.,^37^ where decreased autophagy was observed in Taxol‐treated cells, suggesting that dynamic changes in MT may be more critical for autophagosome generation, whereas MT stabilization limits the increase in the number of autophagosomes.

Notably, depolymerization of MF or MT results in a reduction in the elastic modulus and autophagy triggered by mechanical forces. We inferred that the cytoskeletal network was sparse or incomplete, resulting in diminished function. Therefore, when the cytoskeletal network is incomplete, even a large cell deformation may not be sufficient to complete the entire process, from sensing a mechanical force to the occurrence of autophagy, thus weakening force‐induced autophagy if viewed from a mechanical transduction perspective. This phenomenon strongly suggests that an intact network structure is essential for force‐induced autophagy.

Second, compression at the top of the cell causes deformation of the cell membrane, leading to the cortical MFs (MFs in direct contact with cell membranes) deformation, if the compression is sufficiently large. Mechanical force‐induced cellular deformation is both a sensor of external signals and a transduction pathway,^13^, ^16^, ^38^, ^39^, ^40^, ^41^, ^42^ although the underlying mechanism has not been fully explored.

Taken together, these results indicated that MF is an essential component of mechanoforce‐induced autophagy. The deformation of the cortical MF network may be a key pathway in compression‐induced autophagy (perception, transmission and transduction). Polymerization of the MF network improves cellular elasticity, leading to enhanced sensitivity to compressive stimuli. Thus, small cellular deformations caused by mechanical force could produce significantly increased autophagy. In contrast, the stiffness of MT in the network structure is high,^3^ and most MT are located relatively deep inside the cell, far from the cell membrane. Weak forces or small cellular membrane deformations had little effect on MT geometry. Thus, autophagy arising from MF deformation by compressive forces accounts for a large proportion of these cases.

## 5 CONCLUSION

We evaluated the role of cytoskeletal elements MF and MT in mechanical stress‐induced autophagy using fluorescence imaging and biochemical techniques. Our results suggest that MF is an essential element, whereas MT plays as an auxiliary factor in mechanical stress‐induced autophagy. We infer that the deformation of the elastic cortical MF caused by compression accounts for a large proportion of force‐induced autophagy. Overall, our findings demonstrated that mechanical stress‐induced autophagy is dependent on cytoskeletal transduction.

## AUTHOR CONTRIBUTIONS

Lin Liu and Wei Zheng performed most experiment and analysed data. Yuhui Wei, Qian Li, Nan Chen, Qingling Xia, Xingfei Zhou, Lihua Wang, and Jun Hu provided technical assistance. Bin Li, Yanhong Sun, and Lin Liu designed the experiments and wrote the manuscript.

## CONFLICT OF INTEREST STATEMENT

The authors confirm that there are no conflicts of interest.

## Supporting information


**DATA S1.** Supporting Information.

## Data Availability

All data are available upon reasonable request from the corresponding author with the publication.

## References

[1] Hara T , Nakamura K , Matsui M , et al. Suppression of basal autophagy in neural cells causes neurodegenerative disease in mice. Nature. 2006;441:885‐889. 16625204 10.1038/nature04724

[2] Nakai A , Yamaguchi O , Takeda T , et al. The role of autophagy in cardiomyocytes in the basal state and in response to hemodynamic stress. Nat Med. 2007;13:619‐624. 17450150 10.1038/nm1574

[3] Levine B , Kroemer G . Autophagy in the pathogenesis of disease. Cell. 2008;132:27‐42. 18191218 10.1016/j.cell.2007.12.018 PMC2696814

[4] Yamamoto H , Zhang S , Mizushima N . Autophagy genes in biology and disease. Nat Rev Genet. 2023;24:382‐400. 36635405 10.1038/s41576-022-00562-w PMC9838376

[5] Mizushima N . A brief history of autophagy from cell biology to physiology and disease. Nat Cel Biol. 2018;20:521‐527. 10.1038/s41556-018-0092-5 29686264

[6] Vargas JNS , Hamasaki M , Kawabata T , Youle RJ , Yoshimori T . The mechanisms and roles of selective autophagy in mammals. Nat Rev Mol Cell Biol. 2023;24:167‐185. 36302887 10.1038/s41580-022-00542-2

[7] He W , Peng H , Ma J , et al. Autophagy changes in lung tissues of mice at 30 days after carbon black‐metal ion co‐exposure. Cell Prolif. 2020;53:e12813. 32515860 10.1111/cpr.12813 PMC7377941

[8] Zuo T , Xie M , Yan M , et al. In situ analysis of acupuncture protecting dopaminergic neurons from lipid peroxidative damage in mice of Parkinson's disease. Cell Prolif. 2022;55(4):e13213. 35274781 10.1111/cpr.13213 PMC9055900

[9] Echarri A , Pavón DM , Sánchez S , et al. An Abl‐FBP17 mechanosensing system couples local plasma membrane curvature and stress fiber remodeling during mechanoadaptation. Nat Commun. 2019;10:5828. 31862885 10.1038/s41467-019-13782-2 PMC6925243

[10] Miyano T , Suzuki A , Sakamoto N . Actin cytoskeletal reorganization is involved in hyperosmotic stress‐induced autophagy in tubular epithelial cells. Biochem Biophys Res Commun. 2023;663:1‐7. 37116392 10.1016/j.bbrc.2023.04.070

[11] DuFort CC , Paszek MJ , Weaver VM . Balancing forces: architectural control of mechanotransduction. Nat Rev Mol Cell Biol. 2011;12:308‐319. 21508987 10.1038/nrm3112 PMC3564968

[12] Park JS , Burckhardt CJ , Lazcano R , et al. Mechanical regulation of glycolysis via cytoskeleton architecture. Nature. 2020;578:621‐626. 32051585 10.1038/s41586-020-1998-1 PMC7210009

[13] Sun X , Alushin GM . Cellular force‐sensing through actin filaments. FEBS J. 2023;290:2576‐2589. 35778931 10.1111/febs.16568 PMC9945651

[14] Sun X , Phua DYZ , Axiotakis L Jr , et al. Mechanosensing through direct binding of tensed F‐actin by LIM domains. Dev Cell. 2020;55:468‐482.e7. 33058779 10.1016/j.devcel.2020.09.022 PMC7686152

[15] Schiller HB , Fässler R . Mechanosensitivity and compositional dynamics of cell‐matrix adhesions. EMBO Rep. 2013;14:509‐519. 23681438 10.1038/embor.2013.49 PMC3674437

[16] Romet‐Lemonne G , Jégou A . Mechanotransduction down to individual actin filaments. Eur J Cell Biol. 2013;92:333‐338. 24252518 10.1016/j.ejcb.2013.10.011

[17] Zimmermann D , Kovar DR . Feeling the force: formin's role in mechanotransduction. Curr Opin Cell Bio. 2019;56:130‐140. 30639952 10.1016/j.ceb.2018.12.008

[18] Liu X , Wei Y , Li W , Li B , Liu L . Cytoskeleton induced the changes of microvilli and mechanical properties in living cells by atomic force microscopy. J Cell Physiol. 2021;236:3725‐3733. 33169846 10.1002/jcp.30110

[19] Li B , Wei Y , Li Q , et al. Nanomechanical induction of autophagy‐related fluorescence in single cells with atomic force microscopy. Adv Sci (Weinh). 2021;8:e2102989. 34708576 10.1002/advs.202102989 PMC8693060

[20] Wang K , Wei Y , Liu W , et al. Mechanical stress‐dependent autophagy component release via extracellular Nanovesicles in tumor cells. ACS Nano. 2019;13:4589‐4602. 30884224 10.1021/acsnano.9b00587

[21] Tian T , Sun Y , Wu H , et al. Acupuncture promotes mTOR‐independent autophagic clearance of aggregation‐prone proteins in mouse brain. Sci Rep. 2016;6:19714. 26792101 10.1038/srep19714 PMC4726430

[22] King JS , Veltman DM , Insall RH . The induction of autophagy by mechanical stress. Autophagy. 2011;7:1490‐1499. 22024750 10.4161/auto.7.12.17924 PMC3327616

[23] Kabeya Y , Mizushima N , Ueno T , et al. LC3, a mammalian homologue of yeast Apg8p, is localized in autophagosome membranes after processing. EMBO J. 2000;19:5720‐5728. 11060023 10.1093/emboj/19.21.5720 PMC305793

[24] Flanagan MD , Lin S . Cytochalasins block actin filament elongation by binding to high affinity sites associated with F‐actin. J Biol Chem. 1980;255:835‐838. 7356663

[25] Kruppa AJ , Kendrick‐Jones J , Buss F . Myosins, actin and autophagy. Traffic. 2016;17:878‐890. 27146966 10.1111/tra.12410 PMC4957615

[26] Kast DJ , Dominguez R . The cytoskeleton‐autophagy connection. Curr Biol. 2017;27:R318‐R326. 28441569 10.1016/j.cub.2017.02.061 PMC5444402

[27] Zhao HY , Chen YQ , Luo XY , et al. Ligand phase separation‐promoted, "squeezing‐out" mode explaining the mechanism and implications of neutral nanoparticles that escaped from lysosomes. ACS Nano. 2024;18:2162‐2183. 38198577 10.1021/acsnano.3c09452

[28] Huang Y , Peng Q , Tian X , et al. Nuclear membrane protein SUN2 promotes replication of flaviviruses through modulating cytoskeleton reorganization mediated by NS1. Nat Commun. 2024;15:296. 38177122 10.1038/s41467-023-44580-6 PMC10766649

[29] Yang L , Zhao L , Cui L , et al. Decreased α‐tubulin acetylation induced by an acidic environment impairs autophagosome formation and leads to rat cardiomyocyte injury. J Mol Cell Cardiol. 2019;127:143‐153. 30582931 10.1016/j.yjmcc.2018.12.011

[30] Pollard TD , Cooper JA . Actin, a central player in cell shape and movement. Science. 2009;326:1208‐1212. 19965462 10.1126/science.1175862 PMC3677050

[31] Liu L , Wei Y , Liu J , et al. Spatial high resolution of actin filament organization by PeakForce atomic force microscopy. Cell Prolif. 2020;53:e12670. 31568631 10.1111/cpr.12670 PMC6985672

[32] Saraswathibhatla A , Indana D , Chaudhuri O . Cell‐extracellular matrix mechanotransduction in 3D. Nat Rev Mol Cell Biol. 2023;24:495‐516. 36849594 10.1038/s41580-023-00583-1 PMC10656994

[33] Fletcher DA , Mullins RD . Cell mechanics and the cytoskeleton. Nature. 2010;463:485‐492. 20110992 10.1038/nature08908 PMC2851742

[34] Salbreux G , Charras G , Paluch E . Actin cortex mechanics and cellular morphogenesis. Trends Cell Biol. 2012;22:536‐545. 22871642 10.1016/j.tcb.2012.07.001

[35] Calzado‐Martín A , Encinar M , Tamayo J , Calleja M , San PA . Effect of actin organization on the stiffness of living breast cancer cells revealed by peak‐force modulation atomic force microscopy. ACS Nano. 2016;10:3365‐3374. 26901115 10.1021/acsnano.5b07162

[36] Liu Y , Mollaeian K , Shamim MH , Ren J . Effect of F‐actin and microtubules on cellular mechanical behavior studied using atomic force microscope and an image recognition‐based cytoskeleton quantification approach. Int J Mol Sci. 2020;21:392. 31936268 10.3390/ijms21020392 PMC7014474

[37] Geeraert C , Ratier A , Pfisterer SG , et al. Starvation‐induced hyperacetylation of tubulin is required for the stimulation of autophagy by nutrient deprivation. J Biol Chem. 2010;285:24184‐24194. 20484055 10.1074/jbc.M109.091553 PMC2911293

[38] Hayakawa K , Tatsumi H , Sokabe M . Actin stress fibers transmit and focus force to activate mechanosensitive channels. J Cell Sci. 2008;121:496‐503. 18230647 10.1242/jcs.022053

[39] Massou S , Nunes Vicente F , Wetzel F , et al. Cell stretching is amplified by active actin remodelling to deform and recruit proteins in mechanosensitive structures. Nat Cell Biol. 2020;22:1011‐1023. 32719553 10.1038/s41556-020-0548-2

[40] Chen L , Yu X , Chen W , et al. Nanoscale detection of carbon dots‐induced changes in actin skeleton of neural cells. J Colloid Interface Sci. 2024;668:293‐302. 38678885 10.1016/j.jcis.2024.04.152

[41] Li D , Li J , Hu J , et al. Nanomechanical profiling of Aβ42 oligomer‐induced biological changes in single hippocampus neurons. ACS Nano. 2023;17(6):5517‐5527. 36881017 10.1021/acsnano.2c10861

[42] Yu X , Chen L , Tang M , et al. Revealing the effects of curcumin on SH‐SY5Y neuronal cells: a combined study from cellular viability, morphology, and biomechanics. J Agric Food Chem. 2019;67(15):4273‐4279. 30929442 10.1021/acs.jafc.9b00314

