# Supplementary material for: Mechanical stress‐induced autophagy is cytoskeleton dependent
Source: Cell Prolif. 2024 Aug 18;57(12):e13728. doi: 10.1111/cpr.13728 (PMC11628738; doi:10.1111/cpr.13728)
Supplement: Supplementary file 1 — DATA S1. Supporting Information. [file CPR-57-e13728-s001.docx]

**Supporting Information**

**Mechanical stress-induced autophagy is cytoskeleton dependent**

Lin Liu^1^ | Wei Zheng^2^ | Yuhui Wei^3^ | Qian Li^4^ | Nan Chen^5^ | Qingling Xia^2^ | Lihua Wang^3^ | Jun Hu^3^| Xingfei Zhou^7^ | Yanhong Sun^3*^ | Bin Li^3*^

^1^Key Laboratory of Laboratory Medicine, Ministry of Education of China, Zhejiang Provincial Key Laboratory of Medical Genetics, School of Laboratory Medicine and Life Sciences, Wenzhou Medical University, Wenzhou, 325035*,* China

^2^Shanghai Institute of Applied Physics, Chinese Academy of Sciences, University of Chinese Academy of Sciences, Shanghai, 201800, China

^3^The Interdisciplinary Research Center, Shanghai Synchrotron Radiation Facility, Shanghai Advanced Research Institute, Chinese Academy of Sciences, Shanghai, 201210, China

^4^School of Chemistry and Chemical Engineering, Frontiers Science Center for Transformative Molecules and National Center for Translational Medicine, Shanghai Jiao Tong University, Shanghai, 200024, China

^5^School of Chemistry and Materials Sciences, Shanghai Normal University, Shanghai, 200233, China

^6^Institute of Materiobiology, College of Science, Shanghai University, Shanghai China

^7^ Department of Microelectronic Science and Engineering, School of Physical Science and Technology, Ningbo University, Zhejiang, China

*E-mail: sunyanhong@shu.edu.cn; libin@sari.ac.cn

**MARERIALS AND METHODS**

For Figure S1: the MFs were stained by Phalloidin (Invitrogen). Hoechst 33258 (Invitrogen) was used as a counterstain for the nucleus.

For Figure S3: the MTs were stained by tubulin primary antibodies (Beyotime) overnight at 4 °C, and then FITC-secondary antibodies (Beyotime) for 2 h at room temperature. Hoechst 33258 (Invitrogen) was used as a counterstain for the nucleus.

For Figure S4: the cells fixed with 4% paraformaldehyde were incubated with primary antibodies (α-tubulin (Beyotime), LC3II (Cell Signaling Technology), and p62 primary antibody (Cell Signaling Technology)) overnight at 4 °C, and incubated with Alexa Fluor 647-secondary antibodies (Beyotime) for 2 h at room temperature. DAPI (Beyotime) was used as a counterstain for the nucleus.

For Figure S5: cells were first treated with nocodazole (2 μM) for 120 min; then modiﬁed Eagle’s medium was replaced by L-15 medium; finally, cells were cultured in L-15 medium for 5‒10 min. (C) After the cells were subjected to a stress of 0.52 kPa in L-15 medium for five minutes.

RESULTS


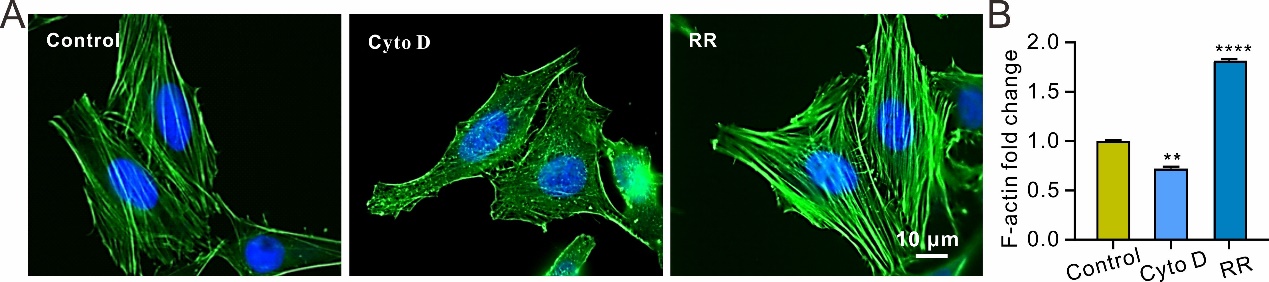


**Figure S1.** Imaging and characterization of MFs in HeLa cells. (A) The change of MFs was detected under Cyto D and RR treatments using a ﬂuorescence microscope. (B) The relative fluorescence intensity of MFs was analyzed by Image J software. ***P* < 0.01, *****P* < 0.0001.


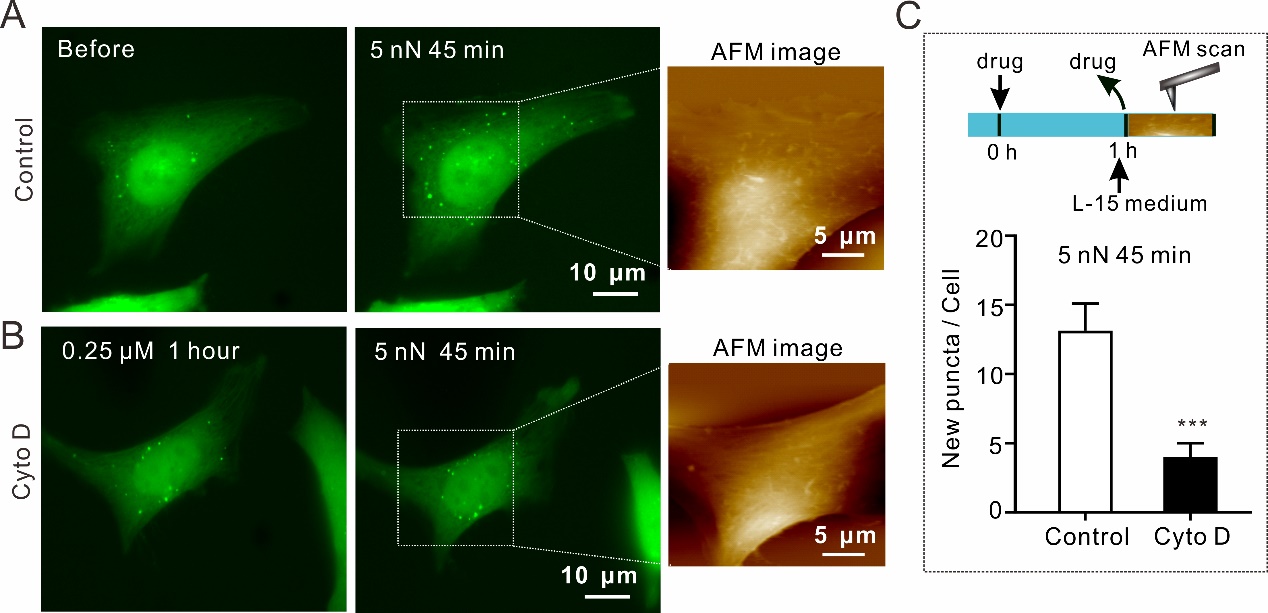


**Figure S2.** Nanomechanical stress-induced autophagy using AFM. (A) Changes in autophagy in HeLa-EGFP-LC3 cells before and after a scanning force with AFM using a 20-nm-diameter probe loaded with 5 nN force lasting 45 min. (left) Fluorescence images before and after the scanning process of the AFM probe. (right) The corresponding AFM image shows the scanning area indicated with the white square. (B) Changes in autophagy in the Cyto D-pretreated HeLa-EGFP-LC3 cells before and after a scanning force with AFM using a 20 nm-diameter probe loaded with 5 nN force lasting 45 min. (left) Fluorescence images before and after the scanning process of the AFM probe (DNP, diameter of 20 nm, nominal 0.35 N/m cantilever). (right) The corresponding AFM image shows the scanning area indicated with the white square. (C) Changes in autophagosome (puncta) in the different groups of cells (n ≥ 3, ^***^*P*<0.001).


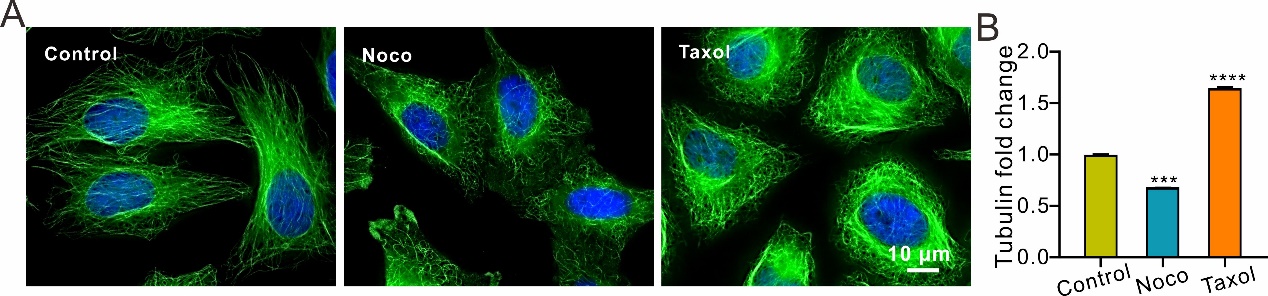


**Figure S3.** Imaging and characerization of MTs in HeLa cells. (A) The change of MTs was detected under nocodazole and Taxol treatments using a ﬂuorescence microscope. (B) The relative fluorescence intensity of MTs was analyzed by Image J software. ^***^*P* < 0.001, ^****^*P* < 0.0001.


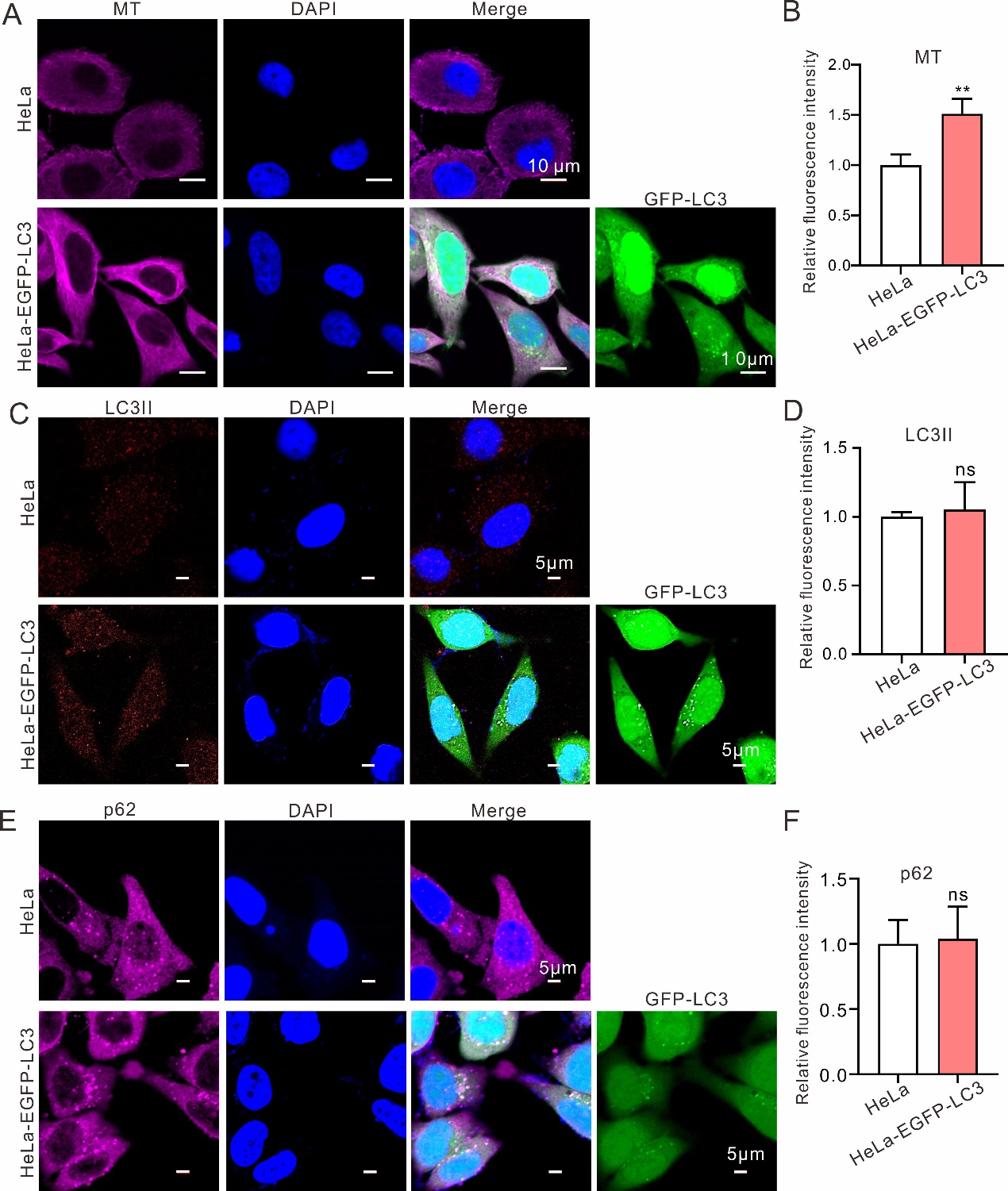


**Figure S4** Imaging and characterization of MTs, LC3II and p62 in HeLa and HeLa-EGFP-LC3 cells. Fluorescence images of MTs (A), LC3II (C) and p62 (E) were obtained using a laser confocal fluorescence microscope. Relative fluorescence intensity of MTs (B), LC3II (D) and p62 (F) analyzed by Image J software. ns, not significant, ^**^*P* < 0.01.

**Figure S5.** Dynamics changes in autophagosomes induced by mechanical stress in HeLa-EGFP-LC3 cells treated with nocodazole. (A) Schematic illustration of the change in EGFP-LC3 puncta after Nocodazol treatments and compression. (B) Dynamics of autophagosomes before or after Nocodazol for 120 min, and 0.52 kPa for 5 min in HeLa-EGFP-LC3 cells.


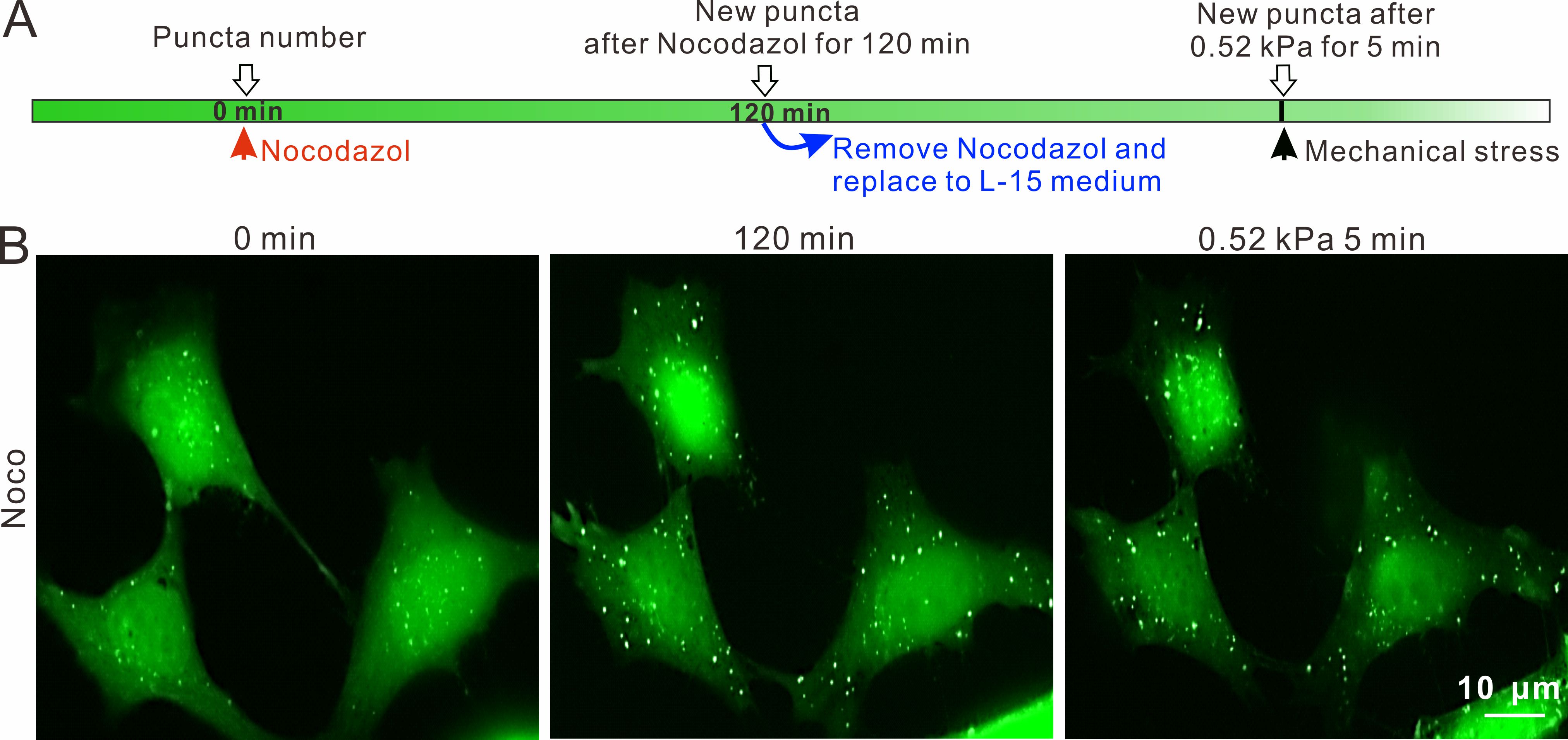


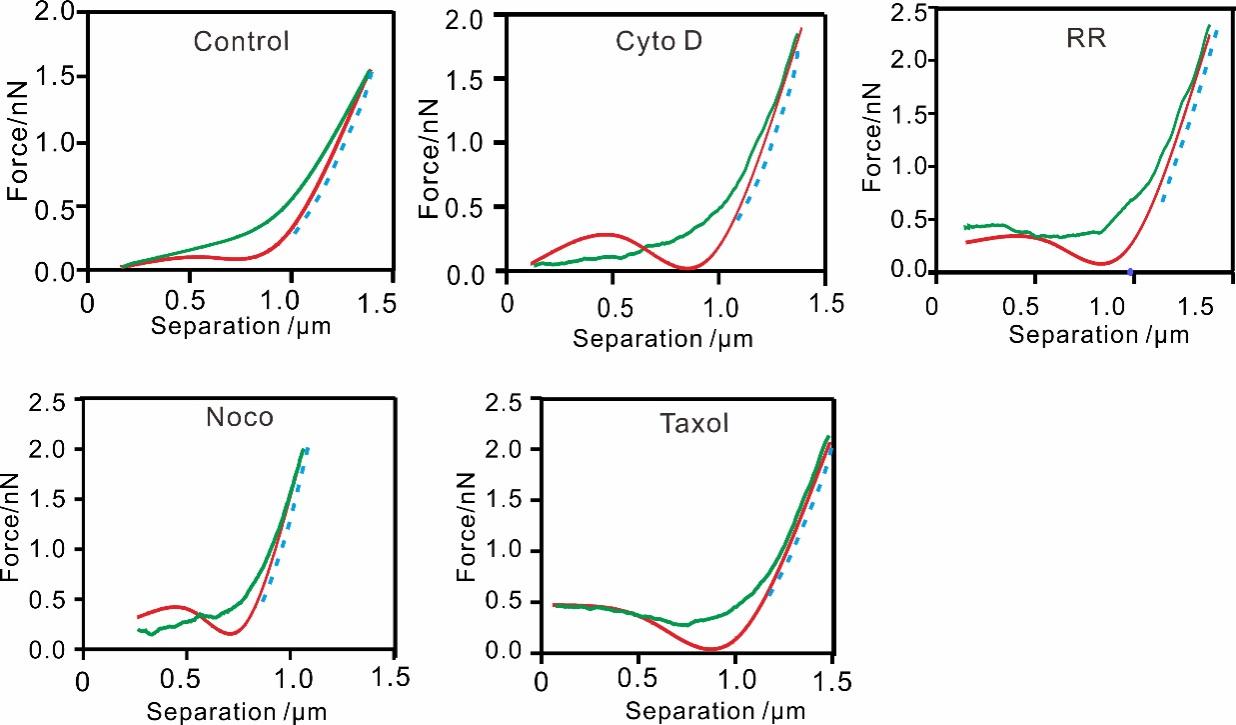


**Figure S6.** Typical force-curves (trace curves in green and retrace curves in red) obtained using AFM with a probe of 2.5-μm-diameter (calculated by manufactory (Novascan Technologies, USA) and a nominal spring constant of 0.35 N/m) loading on the top of HeLa-EGFP-LC3 cells treated with different drugs.
